# Sequencing Illustrates the Transcriptional Response of *Legionella pneumophila* during Infection and Identifies Seventy Novel Small Non-Coding RNAs

**DOI:** 10.1371/journal.pone.0017570

**Published:** 2011-03-03

**Authors:** Barbara A. Weissenmayer, James G. D. Prendergast, Amanda J. Lohan, Brendan J. Loftus

**Affiliations:** 1 UCD Conway Institute for Biomolecular and Biomedical Research, Dublin, Ireland; 2 MRC Human Genetics Unit, Western General Hospital, Edinburgh, United Kingdom; The University of Maryland, United States of America

## Abstract

Second generation sequencing has prompted a number of groups to re-interrogate the transcriptomes of several bacterial and archaeal species. One of the central findings has been the identification of complex networks of small non-coding RNAs that play central roles in transcriptional regulation in all growth conditions and for the pathogen's interaction with and survival within host cells. *Legionella pneumophila* is a Gram-negative facultative intracellular human pathogen with a distinct biphasic lifestyle. One of its primary environmental hosts in the free-living amoeba *Acanthamoeba castellanii* and its infection by *L. pneumophila* mimics that seen in human macrophages. Here we present analysis of strand specific sequencing of the transcriptional response of *L. pneumophila* during exponential and post-exponential broth growth and during the replicative and transmissive phase of infection inside *A. castellanii*. We extend previous microarray based studies as well as uncovering evidence of a complex regulatory architecture underpinned by numerous non-coding RNAs. Over seventy new non-coding RNAs could be identified; many of them appear to be strain specific and in configurations not previously reported. We discover a family of non-coding RNAs preferentially expressed during infection conditions and identify a second copy of 6S RNA in *L. pneumophila*. We show that the newly discovered putative 6S RNA as well as a number of other non-coding RNAs show evidence for antisense transcription. The nature and extent of the non-coding RNAs and their expression patterns suggests that these may well play central roles in the regulation of *Legionella* spp. specific traits and offer clues as to how *L. pneumophila* adapts to its intracellular niche. The expression profiles outlined in the study have been deposited into Genbank's Gene Expression Omnibus (GEO) database under the series accession GSE27232.

## Introduction

*Legionella pneumophila*, the causative agent of Legionnaires disease and Pontiac fever, is commonly found in aquatic habitats where it survives and replicates in protozoa and biofilms [1]. The occurrence of infected amoebae in fresh-water rivers and cooling towers places *Legionella* spp. at the front line of emerging pathogens [2]. *Legionella's* ready-made virulence and lack of person-to-person transmission has led many researchers to believe that the ability of *Legionella* spp. to survive and multiply within macrophages has likely evolved due to a long association with environmental hosts. It is thought that the selective pressure exerted by grazing environmental predators has resulted in an adaption towards bacterial pathogenicity. A primary environmental host for *L. pneumophila* is the free-living amoeba *Acanthamoeba castellanii* and its life cycle within *A. castellanii* mirrors that found in alveolar macrophages [3]. In the biphasic life cycle of *L. pneumophila* the replicative phase (RP) of the bacterium is transitioned to a highly virulent, transmissive phase (TP) [4]. This can be modeled by growing the bacteria in BYE broth, where the exponential phase culture (E) mimics RP, and the post-exponential stationary phase (PE) models TP of the bacteria after infection [5].

The high metabolic rate required in the bacterial replicative phase (RP) is reflected both in the transcriptional and translational responses. As nutrients become limited the pathogen switches to TP resulting in an overall metabolic slow down as the bacterium prepares for host cell egress by biosynthesis and assembly of flagella [5]. The differential gene expression during the switch from RP to TP inside of *A. castellanii* has previously been investigated using *L. pneumophila* microarrays [6]. Half of the *L. pneumophila* genes were shown to be differentially expressed with those involved in energy production and translation strongly down-regulated during TP in favor of those important for intracellular signaling and motility.

Recent sequencing based studies have illuminated increased transcriptional complexity within the genome structures of bacteria and modifications now allow use of strand specific sequencing which provides an accurate determination of the strand of origin of expressed regions of the genome [7], [8], [9], [10]. Through such studies an ever-increasing number of regulatory non-coding RNAs (ncRNAs), typically 100–300 nucleotides in length have been identified both in intergenic regions (IGR) and on the antisense strand of coding sequences. ncRNAs modulate gene expression at the post-transcriptional level through base-pairing with target mRNAs regulating relative levels of translation or decay [11]. In pathogenic bacteria ncRNAs regulate the expression of virulence genes and genes involved in the stress-response important for survival in the host [12].

A feature of these studies is that many of the identified ncRNAs appear strain or species restricted implying that they may serve important functions in species specific traits and virulence properties of pathogenic bacteria. Therefore, in addition to extending previous microarray findings, an aim of this study was to uncover the nature and extent of infection associated ncRNAs through a sequence based whole transcriptome analysis of *L. pneumophila* grown both in BYE broth and under infection conditions in *A. castellanii*.

## Results and Discussion

### Differentially expressed genes of *L. pneumophila* during broth and intracellular growth

Total RNA of *L. pneumophila* Philadelphia-1.pMip-GFP was extracted at various time points during both growth in BYE broth and intracellular growth after infection of *A. castellanii* Neff. The BYE broth growth time points representing exponential stage (E), late exponential stage (LE) and post-exponential stage (PE) cultures were 7, 10 and 12 hours respectively (t7, t10 and t12) (Fig. S1A). Successful infection inside of the amoebae was monitored by viable cell counts and fluorescence microscopy (Fig. S1B and Fig. S2). The infection time points representing the replicative phase (RP) and the virulent, transmissive phase (TP) of the bacteria were 11 and 14 hours respectively (t11 and t14). Differential gene expression was compared between exponential and post-exponential growth in BYE broth and between the replicative and transmissive phase of infection. After DNase treatment and removal of ribosomal RNA, strand specific RNA-seq libraries were generated for each chosen time point and sequencing was carried out on the Illumina GA_IIx_ platform.

**Figure 1:**
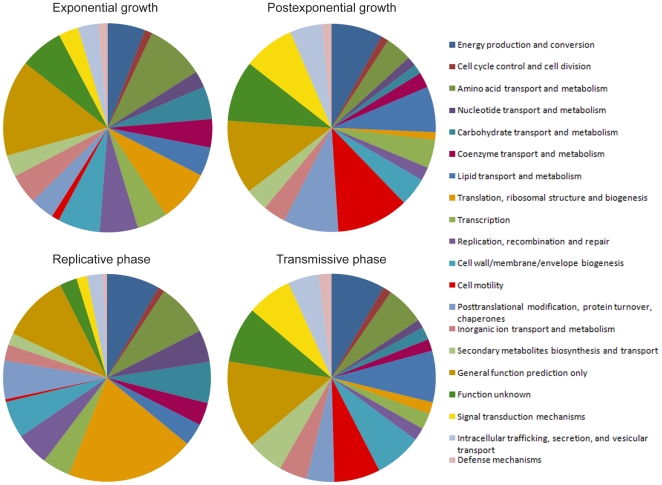
Relative abundance of *L. pneumophila* genes differentially expressed during growth in broth and during intracellular growth in *A. castellanii*. Colors correspond to categories of genes in the COG database. Exponential (E) and post-exponential (PE) growth denotes *L. pneumophila* Philadelphia-1 grown in BYE broth. Replicative phase (RP) and transmissive phase (TP) represents *L. pneumophila* Philadelphia-1 growth inside of *A. castellanii* Neff. In the late growth stages genes coding for proteins important for replication and energy production are replaced by genes coding for proteins involved in cell motility and signal transduction. Additionally in the transmissive phase genes coding for proteins important for lipid transport and metabolism are also strongly up-regulated. The relative gene abundance is listed in Table S4.

Analysis of the differential gene expression of *L. pneumophila* during amoebal infection and growth in BYE broth with a q-value cut off <0.0001 resulted in an up-regulation of 506 genes in TP and 354 genes in PE (Table S1, S2, S3). When we examine the gene expression according to Cluster of Orthologous Groups of proteins (COG) [13] we found that genes coding for proteins involved in translation, ribosomal structure and biogenesis were strongly up-regulated in the replicative phase of infection and its equivalent exponential phase (E) in broth culture (Fig. 1 and Table S4). Transition to TP during infection or PE during broth growth resulted in increased expression of genes associated with cell motility and signal transduction. We also found that genes coding for proteins involved in lipid transport and metabolism were significantly up regulated in TP. Recently it was shown that *L. pneumophila* mutants defective in membrane lipid biosynthesis manifest a poorly functioning Dot/Icm apparatus with low levels of flagellin protein resulting in an unproductive interaction with host cells [14]. Signaling cascades governing flagellum biosynthesis, one of the major virulence traits in TP, have previously been characterized and revealed a complex regulatory interplay between signal transduction and virulence gene expression [15], [16]. In agreement with these findings the flagellar biosynthesis sigma factors *fliA* and *rpoN*, and the stationary phase sigma factor *rpoS* were strongly induced in PE and TP as well as many two-component system regulators (lpg0278, lpg0879, lpg1174, lpg2181, lpg2457, lpg2458, lpg2732). Moreover, stress response genes and virulence genes mediating enhanced entry into macrophages (*enhA/B/C*) were also induced under late infection (TP) and PE conditions. We detected a strong simultaneous up-regulation of genes located on the *L. pneumophila* efflux island (lpg1011–lpg1096), yet it was shown that this metal efflux island is not required for survival of the bacterium in either amoebae or macrophages [17].

Two neighboring clusters, one consisting of genes coding hypothetical proteins (lpg0665–lpg0669) and the other (lpg0670–lpg0674) containing genes coding for a putative hydrolase, a NADH dehydrogenase transmembrane protein, an acetoacetate decarboxylase (ADC), a putative signal peptide and an adenylate cyclase were up-regulated in both PE and TP. During the late stage of infection oxygen availability is limited and NADH dehydrogenase has been suggested as a possible mechanism for coping with changes in oxygen availability [18].

*L. pneumophila* utilizes amino acids as both carbon and energy source and Glutamate serves as the principal energy source whereas glucose has been reported to have no effect on the growth of the bacterium [19]. We could find expression of genes coding for proteins involved in the Entner-Doudoroff pathway, in the Pentose-phosphate pathway, and in glycolysis in both E and RP (Table S5). This data along with a more recent analysis of glucose metabolism in *L. pneumophila* indicate that the bacterium utilizes exogenous glucose and that a functioning Entner-Doudoroff pathway is at least necessary for intracellular growth [20], [21].

### Transmissive phase: Genes significantly up-regulated during infection only

Altogether 144 genes were up-regulated in TP accompanied by a down-regulation in the post-exponential stage in BYE broth (Table S6). We found that known virulence factors including the major *dot*/*icm* gene cluster coding for structural proteins of the type IVB secretion system, and *dotA*, *dotC* and *icmW*, as well as *lssZ*, a structural protein of the type I secretion system, are strongly up-regulated in the TP but not in broth culture (lpg0446, lpg0448–lpg0452, lpg0455, lpg1513, lpg2675, lpg2686, lpg2688). Two genes involved in glutamine biosynthesis and metabolism are up-regulated in TP only (lpg1734, lpg2252) and it has been shown that L-glutamine supports intracellular growth, and may be an substantial energy source in the late stage of the intracellular life cycle [22]. Important transmissive traits include transport of arginine, and L-arginine availability functions as a regulatory signal during *L.pneumophila* intracellular growth [23]. Correspondingly we identified also a strong up-regulation of an arginine ABC transporter in TP accompanied by a down-regulation in PE (lpg0678).

Next to the metal efflux island which is up-regulated in TP and PE we found additional efflux genes and several transporters that are up-regulated in TP but down-regulated in PE (lpg0659, lpg0841, lpg2134, lpg2135, lpg2512, lpg2514). The outer membrane proteins TolC (lpg0827) and OmpA (lpg0657) were both up-regulated in TP and significantly down-regulated in PE. TolC is known to play a crucial role in the *L. pneumophila* virulence probably due to its involvement in efflux pump mechanisms [24]. In other bacteria OmpA is important for stress survival and it has also been shown that an *E. coli* OmpA knockout mutant has a reduced ability to invade *Acanthamoeba* [25]. Pilus assembly (reviewed in Cianciotto 2001 and Molmeret et al 2004 [26], [27]) appears to play a more pronounced role under infection conditions, as the pilus type IV biogenesis and assembly genes (lpg0927, lpg1299) are strongly up-regulated in TP. Furthermore two genes coding for zinc metalloproteases and the gene for the major secreted lysophospholipase A (*plaA*), important for effective host infection, were also strongly up-regulated under infection conditions, and they all have been found in secreted outer membrane vesicles of *L. pneumophila* (lpg0467, lpg2343, lpg2977) [28]. While *plaA* has been shown to detoxify lytic lipids [29], one of the zinc metalloproteases, *proA* (lpg0467) might be involved in several steps of the infection, eg nutrient acquisition, and compromise host defence. Interestingly, *proA* was up-regulated in TP in our study but down-regulated in TP in the microarray study [6]. However, recently Rossier et al found that *proA* might illustrate how the significance of a *Legionella* trait can vary depending on the amoeba host used [30], and in our study a different *A. castellanii* cell line was used than in the microarray study.

### Identification of non-coding RNAs

The role of non-coding RNAs (ncRNAs) during the biphasic lifestyle of *L. pneumophila* remains largely unknown, however the few that have been identified have been found to play important roles in intracellular multiplication within host cells [31], [32]. Therefore in order to detect putative ncRNAs associated with infection, regions of transcription not attributable to known genes were identified (see methods), and in total 98 regions were found (Table S7). The previously identified regulatory RNAs RsmY, RsmZ, and 6S RNA, all of which have been implicated in the virulence of *L. pneumophila*, were detected in this study. RsmY and RsmZ link the LetA/LetS and CsrA regulatory networks and have been directly implicated in both the virulence and transmission of *L. pneumophila* [31], [33]. Single mutants have no impact, but the ΔRsmYZ strain results in a drastic defect in intracellular growth in *A. castellanii* and THP-1 monocyte-derived macrophages. Both RsmY and RsmZ show a fall in expression levels during transition from RP to TP, with RsmY showing a more pronounced decrease. However the expression of RsmY and RsmZ diverge during the later stages of the growth curve. RsmZ shows substantially increased expression and RsmY a modest decrease in expression in PE, highlighting the different paths of these two ncRNAs in response to infection and broth growth conditions (Table S8).

### Identification of a second 6S homolog in *L. pneumophila*

The regulatory RNA 6S RNA is involved in post-transcriptional regulation at late stationary phase via interaction with the RNA polymerase (RNAP) holoenzyme σ^70^ and σ^S^ [34]. A recently identified 6S RNA (*ssrS*) in *L. pneumophila* has been shown to play an important role in the optimization of intracellular multiplication [32]. One unusual finding of this study was the relatively modest number of genes, whose expression levels were modified in the *ssrS* deletion mutant. One possible explanation for this might be the presence of additional copies of 6S RNA as are more commonly found in gram-positive bacteria [35]. A bioinformatics search of the newly characterized ncRNAs highlighted the presence of an additional copy of 6S RNA (6S2 RNA). Interestingly, of the two predicted copies of 6S RNA, 6S2 RNA had by far the highest expression levels showing an average of almost 10-fold greater expression in the infection time points and greater than 30-fold expression during the growth phase time points in comparison to *ssrS* (Table S8). The high expression levels of 6S2 RNA in particular recapitulate the behavior of 6S RNA observed in other published bacterial transcriptome experiments [36], [37].

Similar to the published study *ssrS* underwent an approximate 4-fold increase during TP and remained relatively flat during the later stages of growth in BYE broth. In contrast the 6S2 RNA showed only slight up-regulation in TP but achieves a very high level of expression during E and PE without any major change in expression. In *Bacillus subtilis* the two copies of 6S RNA are found to show different temporal expression patters in a growth phase dependent manner [35]. This study shows a similar independent timing in expression between the *L. pneumophila* 6S RNA homologs implying a functional divergence and suggesting a different regulatory mechanism in operation during the process of infection than in broth growth.

**Figure 2:**
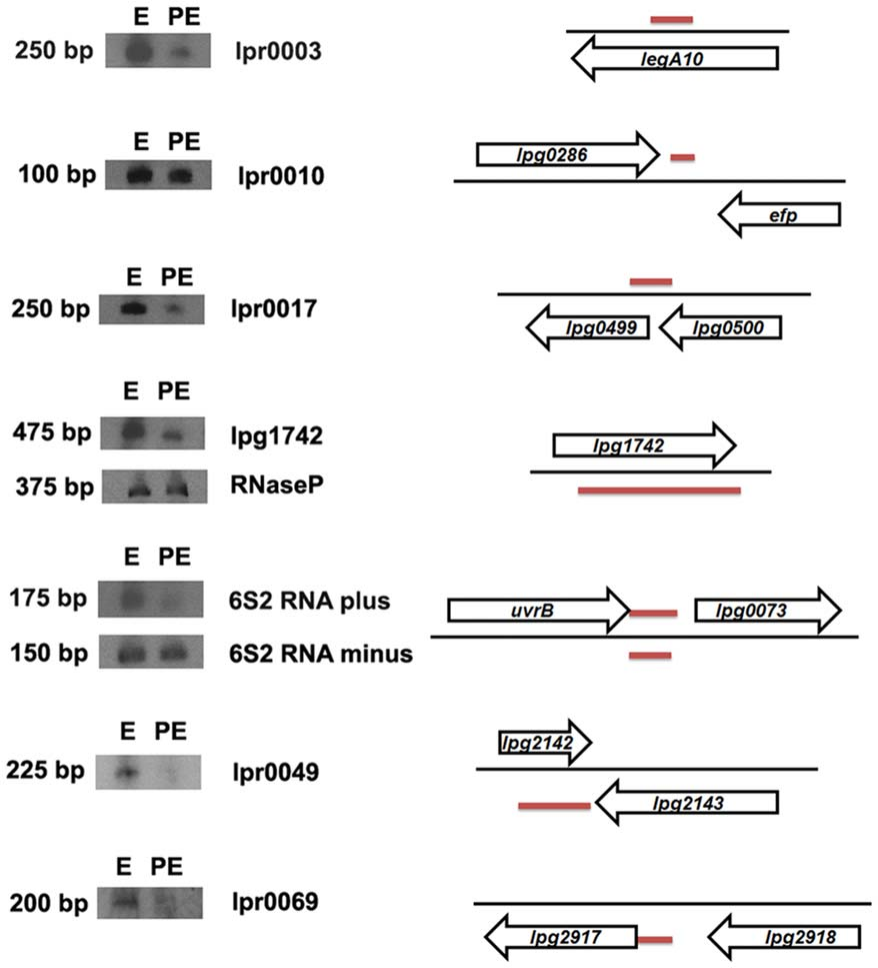
Northern Blot analysis of 8 putative ncRNAs and RNaseP. Equal amounts per lane of *L. pneumophila* Philadelphia-1 total RNA after bacterial growth in BYE broth were electrophoresed on 6% TBE/urea gels and blotted membranes were probed with single-stranded DIG-labeled RNA probes. lpr0049 and lpr0069 represent ncRNA family members. E = exponential growth phase, PE = post-exponential growth phase. Black arrow: gene, red line: ncRNA.

The strand specificity of the sequencing showed evidence for both sense and antisense transcripts at the 6S2 RNA locus, an observation subsequently confirmed using Northern Blots (Fig. 2). The role of the antisense transcripts remains unclear but the palindromic nature of the 6S RNA would be predicted to provide them with significantly stable secondary structures.

Transfer-messenger RNA (tmRNA) is a small regulatory RNA, ubiquitous in bacteria with dual tRNA and messenger RNA like properties that interacts with translating ribosomes in a reaction known as trans-translation [38]. tmRNA along with RNaseP, a regulatory RNA previously characterized in other bacteria, were detected and their elevated expression levels mirrored that found in other studies [36], [37]. As the two 6S RNAs, tmRNA was highly expressed consistent with its role in recovery from a variety of stresses and interestingly both, 6S2 RNA and tmRNA, showed evidence of antisense transcription (Table S8). Interestingly the ribozyme RNaseP involved in cleaving off precursor sequences from tRNA molecules was also accompanied by antisense transcription. The RNaseP ncRNA was located anti-sense to the hypothetical lpg1742 gene. Confirmation of the expression of both RNaseP and lpg1742 was carried out by Northern Blot (Fig. 2).

### Novel non-coding RNAs

Of the remaining 90 transcriptional units 18 showed strong homology to known protein coding genes with the majority matching genes from other *Legionella* spp., suggesting they likely corresponded to mis- or unannotated genes from the *L. pneumophila* Philadelphia-1 genome [39]. The residual newly discovered 72 transcriptional units consequently represent candidate ncRNAs and the expression of a subset was confirmed by Northern Blotting (Table S9, and Fig. 2). Within this remaining set we looked for significantly stable secondary structures, which are displayed by many known functional ncRNAs. Of the 72, 20 (28%) had a predicted secondary structure more stable than at least 95% of 1000 randomly permuted sequences of the same length and base composition (Table S7). These 20 ncRNAs are likely under selection at the DNA level to maintain a stable secondary structure, and consequently represent strong candidates for being functional. Surprisingly one candidate ncRNA (lpr0010) had a predicted secondary structure that was less stable than all 1000 permutations predicted at both 20°C and 37°C. This candidate ncRNA consequently appears to also be evolving non-neutrally, though maintaining an unstable secondary structure. Whether this is a consequence of other pressures at the DNA level is unclear, though the sequence shows no substantial bias in base composition.

### Conservation of ncRNAs across bacterial species

Of the 72 ncRNAs only 12 had homologous sequences found outside other sequenced *L. pneumophila* strains (i.e. Paris, Corby and Lens, ). This finding is unsurprising as there is limited sequence conservation across structured RNAs in different species making them more difficult to identify [36], [40]. Despite the relatively high nucleotide-level conservation between sequenced *L. pneumophila* strains two of the stable ncRNAs (lpr0046 and lpr0048) are both located on the 65 kb pathogenicity island which is specific to the *L. pneumophila* Philadelphia-1 strain [41]. lpr0046 was up-regulated in TP but not in PE, and lpr0048 showed very limited expression in either of the BYE broth times sampled but had a high expression value when grown intracellularly in *A. castellanii* (Table S9). These findings underlie the link between ncRNA expression and virulence as well as their role as putative strain specific markers.

### Many of the identified ncRNAs are antisense to known genes

The majority of regulatory RNAs modulate expression of target mRNAs via base pairing as either cis-encoded antisense RNAs from the opposite strand to protein coding genes or as trans-encoded RNAs from loci unlinked to their targets [42]. In our study 33 of the candidate ncRNAs are expressed, at least partially, antisense to known protein coding genes (Table S10).

There are a number of ncRNAs antisense to genes with relevance to pathogenesis including an outer membrane protein (lpg2961) and a homolog of the structural toxin protein RtxA (lpg0644) which has been shown to be involved in intracellular survival and trafficking in amoebae [43]. Another important virulence gene antisense to a predicted ncRNA is *sdeA* (lpg2157), a member of the SidE family, secreted by the Dot/Icm system during macrophage infection in an IcmS-dependent manner [44]. Other genes that might be important for the survival and virulence of *L. pneumophila* with antisense ncRNAs are the cytochrome D ubiquinol oxidase (lpg1202) [45], NAD-glutamate dehydrogenase (lpg0245) [46] and *Legionella* spp. restricted virulence region protein LvrA (lpg1259) [47]. Moreover, a number of coding genes with antisense ncRNAs are involved in replication, recombination and repair like subunits of DNA topoisomerase IV (lpg0691) and the excinuclease ABC A subunit (lpg0384).

Interestingly, the *Legionella* spp. restricted gene *legA10* (lpg0038) coding for a eukaryotic like ankyrin repeat protein has two antisense ncRNAs on the opposite strand (*lpr0003*/*lpr0004*) and the expression of the coding gene is down-regulated in TP and PE. In contrast, both antisense transcripts are down-regulated in PE but significantly up-regulated in TP. However *lpr003* is expressed at greater than 10 times the level of *lpr004* during infection reducing to a 2-fold difference during growth in BYE broth.

Similar to *Listeria monocytogenes* and *Helicobacter pylori* many of the antisense ncRNAs overlap 5′UTRs and 3′UTRs of coding genes and it is likely that these ncRNAs are RNA regulators of the corresponding gene ([36], and recently reviewed in [48]).

### ncRNAs transcribed antisense to other ncRNAs

There are few published reports of ncRNAs showing significant overlap to other ncRNAs and identification of distinctive ncRNAs ‘pairs’ can sometimes be problematic as a number of ncRNAs overlap with 5′UTRs and 3′UTRs of coding genes. However, we could identify a number of examples of pairs of ncRNAs (6S2 RNA, tmRNA, lpr0007/lpr0008, lpr0022/lpr0023, lpr0041/lpr0042, lpr0055/lpr0056, lpr0060/lpr0061) distinct from coding regions and arising from different strands of the same chromosomal locus. These data demonstrate that such ncRNA “pairs” appear to be a feature of the *L. pneumophila* Philadelphia-1 transcriptome and may offer an additional regulatory layer affecting gene regulation. The finding that the 6S2 ncRNA identified in this study is almost completely overlapped by another ncRNA candidate on the opposite strand offers the intriguing possibility that its regulatory function is in part controlled by its antisense ncRNA.

### Discovery of an ncRNA family that shows differential expression during infection

**Figure 3:**
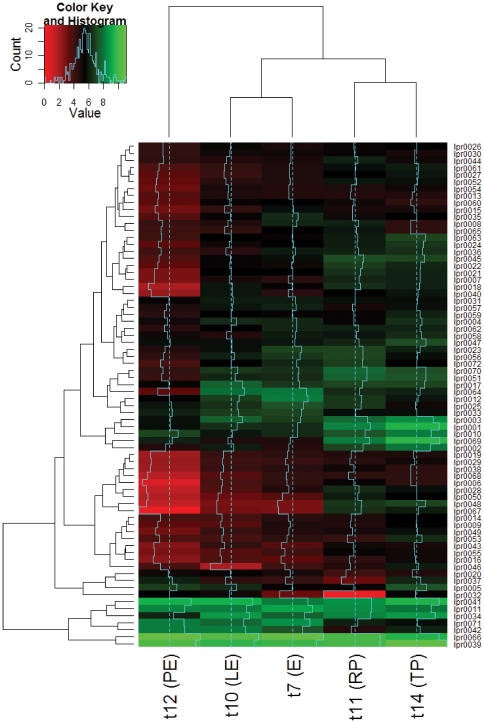
Expression levels of the putative ncRNAs across time points and growth conditions. Expression levels are represented by log2(RPKM) values. Growth time points of *L. pneumophila* Philadelphia-1 grown in BYE broth are t7 (E), t10 (LE) and t12 (PE). Growth time points of *L. pneumophila* Philadelphia-1 inside of *A. castellanii* Neff are t11 (RP) and t14 (TP).

**Figure 4:**
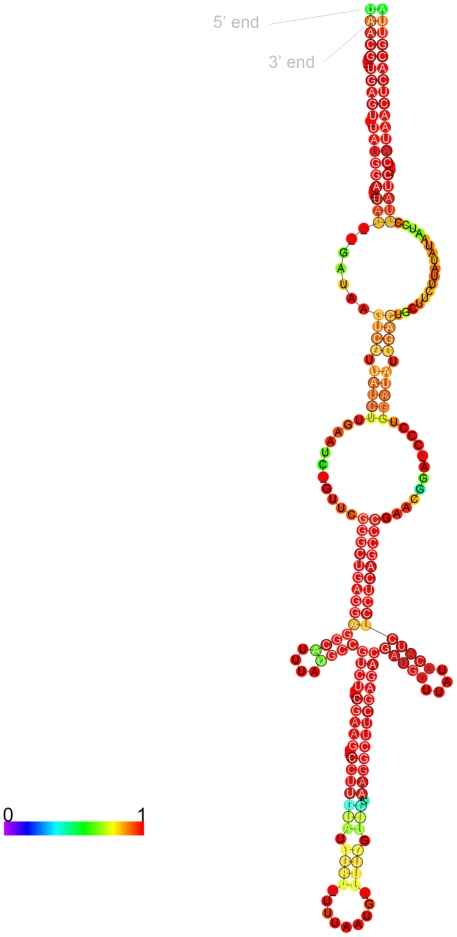
Consensus secondary structure of multi-copy family of infection associated ncRNAs obtained using RNAalifold (PMID: 19014431).

To look for modulators of gene expression specific to infection we searched for a subset of ncRNAs showing infection specific or preferential expression. In total 10 ncRNAs showed average RPKM values at least 6 times higher in infection than seen in *L. pneumophila* grown in broth (Fig. 3) and three of these (lpr0001, lpr0053, and lpr0069) preferentially associated with the transmissive phase of infection had expression levels substantially higher at TP in comparison to RP (Table S9). lpr0001 and lpr0069 displayed substantial levels of homology to one another at both the sequence and structural level and searches highlighted a number of further copies of this sequence throughout the genome. In total 20 homologous sequences could be identified in the *L. pneumophila* Philadelphia-1 genome. The highly stable consensus secondary structure of this family of ncRNAs is evident (Fig. 4) and the expression of two members (lpr0049 and lpr0069) was confirmed by Northern Blot (Fig. 2). The majority of expression arose from lpr0001 or lpr0069, though a number of other family members show relatively limited expression levels (Table S11). A number of copies contain large deletions however no obvious relationship between expression levels and sequence conservation could be identified.

**Figure 5:**
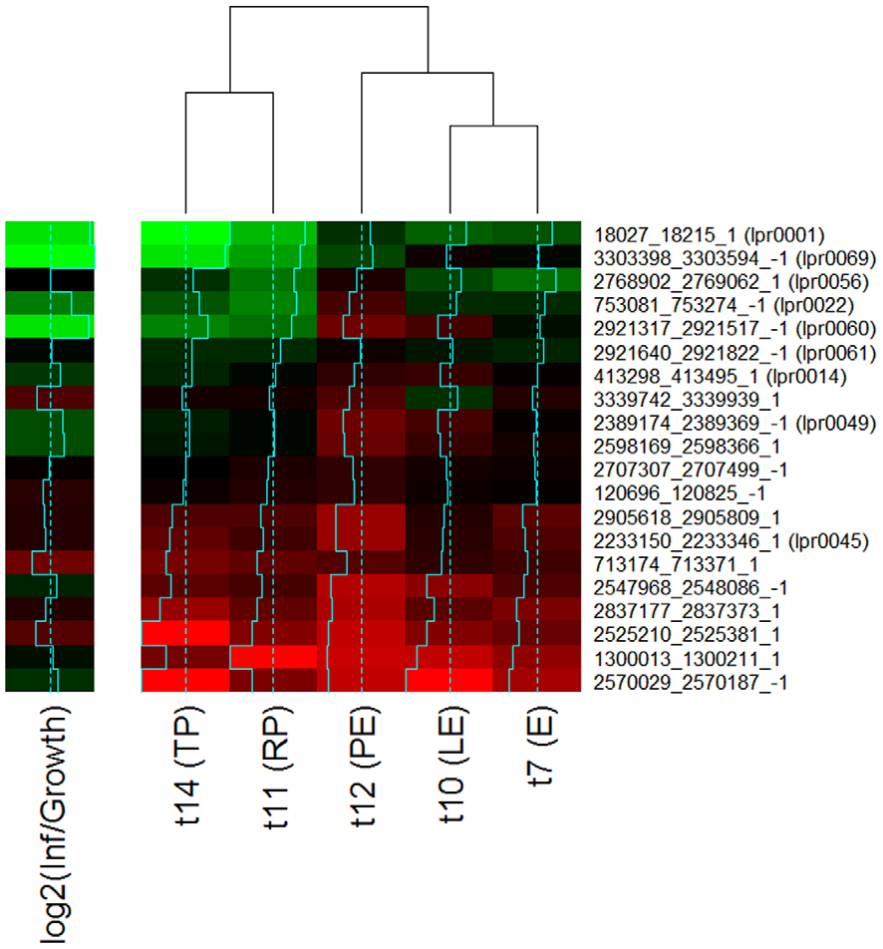
Expression levels of the putative ncRNA family members across time points and growth conditions. Coordinates and strands are shown on the right and correspond to the positions of the core homologous regions shared between copies. Expression levels are represented by log2(RPKM) values. The log ratios of average expression levels in infection versus growth are also shown. Growth time points of *L. pneumophila* Philadelphia-1 grown in BYE broth are t7 (E), t10 (LE) and t12 (PE). Growth time points of *L. pneumophila* Philadelphia-1 inside of *A. castellanii* Neff are t11 (RP) and t14 (TP).

The multiplicity and high sequence conservation across this ncRNA family is reminiscent of the Qrr sRNA family, which controls quorum sensing and virulence in *Vibrio* spp. [49], [50]. *L. pneumophila* shares a quorum sensing capability with *Vibrio* spp., yet there is no evidence that they share analogous roles. However the ncRNA family and the LqsS/LqsR two-component system underlying quorum sensing in *L. pneumophila* are both absent in *L. longbeachae* indicating that the family has diverged or that it is linked to a particular environmental niche. Although the ncRNA family members share a high degree of sequence homology the expression patterns of individual members appears distinctly uncoordinated with each member displaying unique expression profile in the datasets shown (Fig. 5). One member of the ncRNA family is transcribed antisense to a gene coding for a hypothetical protein (lpg2142), whose expression increased during infection relative to growth in broth, implying a potential role in its regulation, though further investigation is required.

To look at the potential distribution of ncRNA family like sequences across a number of other *Legionella* spp. genomes we searched for homologous sequences in the *L. pneumophila* Paris, Alcoy and Corby strains as well as *L. drancourtii* and *L. longbeachae*. Homologous sequences appear in similar configurations and in many cases flanked by similar genes in the genomes of the other *L. pneumophila* strains. No homologous sequences could be found in *L. longbeacheae* and only one could be identified in *L. drancourtii* although this may be due to divergence or the incomplete nature of the genome.

The study of *L. pneumophila* grown in BYE broth or intracellularly in amoebae confirms that in the bacterial biphasic life cycle genes necessary for replication are replaced by those important for cell motility and intracellular signaling in the late stages of infection or growth in broth. A number of virulence factors and transporters were strongly up-regulated particularly in the late infection stage. The high resolution achievable by sequencing revealed that nearly the entire bacterial genome was expressed (Fig. S3) regardless of the bacterial life cycle or growth conditions. We could identify 72 new ncRNAs, some of them differentially expressed in a growth-phase dependent manner; some are differentially expressed under infection conditions only, leading to the assumption that ncRNAs are significant contributors of the *L. pneumophila* pathogenicity. In addition we identified a second putative 6S RNA copy and a number of ncRNAs that form antisense pairs. Finally we could detect a family of ncRNAs consisting of 20 members, which are preferentially expressed during the *L. pneumophila* intracellular life cycle and appear to be present in other clinically important *Legionella* strains (Fig. S4).

## Materials and Methods

### Strains, cell lines and media

*Legionella pneumophila* Philadelphia-1 WT and the constitutively GFP-expressing bacterial strain *Legionella pneumophila* Philadelphia-1 pMip.gfp ( both kindly provided by Antje Flieger, Wernigerode, Germany, [51]) were cultured on buffered charcoal-yeast extract agar (BCYE, Sigma-Aldrich, Gillingham, UK) for 3 days at 37°C (15) or grown in buffered yeast extract (BYE) broth at 37°C with shaking at 250 rpm. Bacterial growth was monitored at an optical density of 600 nm (OD_600_) after inoculation to an OD_600_ of 0.1. *Acanthamoeba castellanii* Neff (ATCC 30010) was cultured in PYG medium (Formedium, Norfolk, UK) at 30°C.

### Intracellular growth of *L. pneumophila* in *A. castellanii*

The infection assays were performed according to Moffat and Tompkins [52]. In brief *A. castellanii* cells were washed in A. c. buffer and adjusted to 10^6^ cells per mL. 10 mL amoebal suspension was transferred to a 75 cm^2^ tissue culture flask and incubated at 37°C for 1 h. Stationary phase *L. pneumophila* Philadelphia-1 grown on BCYE agar, diluted in A.c. buffer, were mixed with *A. castellanii* at a MOI of 100, defining the start point of the time-course experiment. Subsequent to invasion for 1 h at 37°C *A. castellanii* cells were washed three times to remove external bacteria. The infection was monitored by fluorescence microscopy and viable cell counts of *L. pneumophila* on BCYE agar.

### RNA extraction

Total RNA was extracted by resuspending the amoebae in RLT buffer (Qiagen, Hilden, Germany). For efficient cell lysis the suspension was passed 7 times through a 23G needle, and centrifuged at 6000 g for 2 min. The resultant bacterial pellet was resuspended in TE/Lysozyme (1 mg/mL), incubated for 5 min at room temperature, and bacterial RNA was extracted using the Qiagen RNeasy Minikit. DNase treatment was carried out using recombinant DNase from USB (USB Molecular Biology Reagents, High Wycombe, UK), and bacterial and eukaryotic rRNA were removed using the RiboMinus Kit (Invitrogen, Carlsbad, CA, USA).

### Northern Blots

Total RNA of *L. pneumophila* Philadelphia-1 grown in BYE broth was isolated using the RNeasy Mini Kit (Qiagen, Hilden, Germany), and equal amounts of RNA per lane were electrophoresed on 6% TBE/urea gels and blotted onto nylon membranes. Hybridizations were carried out with single-stranded RNA probes, generated via T7 polymerase mediated *in vitro* transcription of PCR products in the presence of DIG-UTP (Oligos used are listed in ). Membranes were stained with CSPstar as per manufacturer's instructions (all reagents were from Roche Molecular Diagnostics, Mannheim, Germany).

### Library generation and RNA-sequencing

Strand specific RNA-seq libraries were prepared for the Illumina GA_IIx_ (Illumina Inc, San Diego, Ca, USA) using a variation on a previously published protocol [53]. Adjustments to the published protocol included alteration in fragmentation, cDNA synthesis and adapter ligation. The DNase treated and rRNA depleted RNA from each time-point was fragmented using divalent cations (Fragmentation Buffer, Ambion Austin, Tx, USA) — 70°C for 5 min — yielding an average size of 200 nucleotides (nt). RNA fragments were precipitated with ethanol. All enzymes and reaction components for cDNA synthesis were obtained from Invitrogen (Carlsbad, CA, USA): first strand cDNA synthesis was carried out in a reaction containing Super Script II reverse transcriptase (200 units), random hexamer primers (3 µg) and dNTPs (500 µM). First strand reaction components were removed with Illustra MicroSpin G-50 columns (GE Healthcare Biosciences, Pittsburg, PA, USA). dUTP containing second strand cDNA was generated with DNA Polymerase I (50 units) and RNaseH (2 units) in a 1 times 2^nd^ strand buffer (Invitrogen, Carlsbad, CA, USA) also containing 300 µM of dATP, dCTP, dGTP and dUTP but no dTTP. Products were further processed, end repaired, addition of a single A to the 3′ end and ligation of indexed adapters. To allow multiplexing of samples 6-nt barcoded Illumina compatible adapters were utilized in place of the commercially supplied adapters [54]. Libraries were sized selected (cut at 200+50 nt) on 2.5% TAE agarose gels. Library material was isolated from the agarose cuts with QiaQuick MinElute Gel Extraction kit (Qiagen, Hilden, Germany). Finally prior to library amplification the dUTP containing second strand was removed via digestion with Uracil DNA Glycoylase (1 unit) (Bioline, London, UK). Purified libraries were quantified using a Qubit™ fluorometer (Invitrogen, Carlsbad, CA, USA) and a Quant-iT™ double-stranded DNA High-Sensitivity Assay Kit (Invitrogen, Carlsbad, CA, USA). Clustering and sequencing of the material was carried out as per manufacturer's instructions – v2 Single Read Cluster Kits and v3 SBS kits (Illumina Inc, San Diego, Ca, USA) were utilized for all sequencing.

### Read mapping

In total 95,731,916 single end reads were generated corresponding to over 3.13 Gb of sequence. Reads were mapped to the *L. pneumophila* Philadelphia-1 genome (NC_002942.5) using the Bowtie short read aligner (PMID: 19261174) with the “–best” flag. In total 52,711,055 reads were successfully mapped to the 3.4 Mb *L. pneumophila* Philadelphia-1 genome, corresponding to a genome-wide average coverage of 560 reads per base pair (Fig. S3). As expected, the majority of unmapped reads came from the infection time points and corresponded to *A.castellanii* transcripts (results not shown). Sam output from bowtie was converted to binary bam files using samtools (PMID: 19505943). Bam files and sequence coverage were visualized using Artemis v12 (PUBMED: 11120685).

### Identification of infection associated novel transcriptional units

Evidence of transcribed ncRNAs was identified by manual inspection of the infection data sets using the Sanger Artemis DNA sequence viewer in conjunction with bam and coverage files. Boundaries of transcribed units were determined using a sliding window of 50 bp to optimize sub-region continuity of expression and TU's (transcriptional units) required a minimum of 20 reads. To prevent misclassification of untranslated regions (UTRs) as ncRNAs, candidates with expression levels broadly similar to an adjacent gene were discarded. Putative unannotated protein coding genes were characterized using Coding Potential Calculator (PMID: 17631615) and each TU was compared to the Rfam (PMID: 18953034) database to characterize any copies of RNA homologous to those previously identified.

### Differential expression

Read counts and Reads Per Million (RPM) values which represent expression values correcting for the numbers of sequence generated were calculated for each gene and novel transcriptional unit using only reads falling entirely within the respective region (and on the correct strand). In the case where different transcripts from the same sample were compared the metric RPKM or Reads Per Kilobase per Million reads was used. RPKM is the same as RPM except that the length of the transcript is also corrected for. All reads mapping to the 16S or 23S ribosomal RNAs were excluded. Differential expression was calculated from raw read counts using the MATR method implemented in the DEGseq R package (PMID: 19855105). Transcript units with a q<0.0001 were deemed differentially expressed. Enrichment of COG terms among genes differentially expressed between time points was tested via the calculation of cumulative hypergeometric P values for each COG term. Genes with a differential expression q value less than 0.001 were compared to the background list of all genes in this analysis. Only enrichment p values exceeding 0.0022 were deemed significant (corresponding to a Bonferroni corrected p value of 0.05 across 23 tested COG terms). Expression differences were measured using log2 fold change (M).

### Secondary structure

Putative secondary structures were characterized using the Vienna RNA package (PMID: 16452114). The significance of the stability of secondary structures was tested via permuting the sequence of the given RNA 1000 times and recalculating the minimum free energy. The number of permutations that exceeded the observed MFE of the real sequences was then calculated. Z scores were also determined for each sequence based on the permutation results. The consensus secondary structure of the described ncRNA family was determined using RNAalifold (PMID: 12079347). The second copy of 6S (6S2) was calculated using the Rfam sequence search facility (http://rfam.sanger.ac.uk/) (PMID: 18953034) and produced an E value of 2.454e-15.

### Homolog identification

Putative paralogs and orthologs of each novel transcriptional unit were identified via blastn comparison of each sequence to all sequenced bacterial genomes. Only hits with an E value<0.001 were retained.

## Supporting Information

Table S1 **Expression counts of the transcriptome.**
(XLS)

Table S2 **Coding genes up-regulated in TP.**
(XLS)

Table S3 **Coding genes up-regulated in PE.**
(XLS)

Table S4 **COG enrichment.**
(XLS)

Table S5 **Coding genes expression for glucose utilization.**
(XLS)

Table S6 **Coding genes up-regulated in TP only.**
(XLS)

Table S7 **ncRNA data sheet.**
(XLS)

Table S8 **Expression counts of known ncRNAs.**
(XLS)

Table S9 **Expression counts of new ncRNAs.**
(XLS)

Table S10 **Antisense ncRNAs.**
(XLS)

Table S11 **Expression counts ncRNA family.**
(XLS)

Table S12 **Oligos used for Northern Blots.**
(XLS)

Figure S1 ***L. pneumophila*** **growth curve in BYE broth and intracellular.**
(DOC)

Figure S2 **GFP-expressing** ***L. pneumophila*** **inside of** ***A. castellanii*** **.**
(DOC)

Figure S3 **Read coverage curve.**
(DOC)

Figure S4 **Trimmed alignment of the non coding RNA family (family A).**
(DOC)

## References

[1] Taylor M, Ross K, Bentham R (2009). Legionella, protozoa, and biofilms: interactions within complex microbial systems.. Microb Ecol.

[2] Berk SG, Gunderson JH, Newsome AL, Farone AL, Hayes BJ (2006). Occurrence of infected amoebae in cooling towers compared with natural aquatic environments: implications for emerging pathogens.. Environ Sci Technol.

[3] Swanson MS, Hammer BK (2000). Legionella pneumophila pathogesesis: a fateful journey from amoebae to macrophages.. Annu Rev Microbiol.

[4] Molofsky AB, Swanson MS (2004). Differentiate to thrive: lessons from the Legionella pneumophila life cycle.. Mol Microbiol.

[5] Molofsky AB, Swanson MS (2003). Legionella pneumophila CsrA is a pivotal repressor of transmission traits and activator of replication.. Mol Microbiol.

[6] Bruggemann H, Hagman A, Jules M, Sismeiro O, Dillies MA (2006). Virulence strategies for infecting phagocytes deduced from the in vivo transcriptional program of Legionella pneumophila.. Cell Microbiol.

[7] Wurtzel O, Sapra R, Chen F, Zhu Y, Simmons BA (2010). A single-base resolution map of an archaeal transcriptome.. Genome Res.

[8] Passalacqua KD, Varadarajan A, Ondov BD, Okou DT, Zwick ME (2009). Structure and complexity of a bacterial transcriptome.. J Bacteriol.

[9] Albrecht M, Sharma CM, Reinhardt R, Vogel J, Rudel T (2010). Deep sequencing-based discovery of the Chlamydia trachomatis transcriptome.. Nucleic Acids Res.

[10] Perkins TT, Kingsley RA, Fookes MC, Gardner PP, James KD (2009). A strand-specific RNA-Seq analysis of the transcriptome of the typhoid bacillus Salmonella typhi.. PLoS Genet.

[11] Frohlich KS, Vogel J (2009). Activation of gene expression by small RNA.. Curr Opin Microbiol.

[12] Romby P, Vandenesch F, Wagner EG (2006). The role of RNAs in the regulation of virulence-gene expression.. Curr Opin Microbiol.

[13] Tatusov RL, Galperin MY, Natale DA, Koonin EV (2000). The COG database: a tool for genome-scale analysis of protein functions and evolution.. Nucleic Acids Res.

[14] Conover GM, Martinez-Morales F, Heidtman MI, Luo ZQ, Tang M (2008). Phosphatidylcholine synthesis is required for optimal function of Legionella pneumophila virulence determinants.. Cell Microbiol.

[15] Hovel-Miner G, Pampou S, Faucher SP, Clarke M, Morozova I (2009). SigmaS controls multiple pathways associated with intracellular multiplication of Legionella pneumophila.. J Bacteriol.

[16] Albert-Weissenberger C, Sahr T, Sismeiro O, Hacker J, Heuner K (2010). Control of flagellar gene regulation in Legionella pneumophila and its relation to growth phase.. J Bacteriol.

[17] Kim EH, Charpentier X, Torres-Urquidy O, McEvoy MM, Rensing C (2009). The metal efflux island of Legionella pneumophila is not required for survival in macrophages and amoebas.. FEMS Microbiol Lett.

[18] Cazalet C, Rusniok C, Bruggemann H, Zidane N, Magnier A (2004). Evidence in the Legionella pneumophila genome for exploitation of host cell functions and high genome plasticity.. Nat Genet.

[19] Keen MG, Hoffman PS (1984). Metabolic pathways and nitrogen metabolism in Legionella pneumophila.. Curr Microbiology.

[20] Harada E, Iida K, Shiota S, Nakayama H, Yoshida S (2010). Glucose metabolism in Legionella pneumophila: dependence on the Entner-Doudoroff pathway and connection with intracellular bacterial growth.. J Bacteriol.

[21] Eylert E, Herrmann V, Jules M, Gillmaier N, Lautner M (2010). Isotopologue profiling of Legionella pneumophila: role of serine and glucose as carbon substrates.. J Biol Chem.

[22] Wieland H, Ullrich S, Lang F, Neumeister B (2005). Intracellular multiplication of Legionella pneumophila depends on host cell amino acid transporter SLC1A5.. Mol Microbiol.

[23] Hovel-Miner G, Faucher SP, Charpentier X, Shuman HA (2010). ArgR-regulated genes are derepressed in the Legionella-containing vacuole.. J Bacteriol.

[24] Ferhat M, Atlan D, Vianney A, Lazzaroni JC, Doublet P (2009). The TolC protein of Legionella pneumophila plays a major role in multi-drug resistance and the early steps of host invasion.. PLoS One.

[25] Alsam S, Jeong SR, Sissons J, Dudley R, Kim KS (2006). Escherichia coli interactions with Acanthamoeba: a symbiosis with environmental and clinical implications.. J Med Microbiol.

[26] Cianciotto NP (2001). Pathogenicity of Legionella pneumophila.. Int J Med Microbiol.

[27] Molmeret M, Bitar DM, Han L, Kwaik YA (2004). Cell biology of the intracellular infection by Legionella pneumophila.. Microbes Infect.

[28] Galka F, Wai SN, Kusch H, Engelmann S, Hecker M (2008). Proteomic characterization of the whole secretome of Legionella pneumophila and functional analysis of outer membrane vesicles.. Infect Immun.

[29] Flieger A, Neumeister B, Cianciotto NP (2002). Characterization of the gene encoding the major secreted lysophospholipase A of Legionella pneumophila and its role in detoxification of lysophosphatidylcholine.. Infect Immun.

[30] Rossier O, Dao J, Cianciotto NP (2008). The type II secretion system of Legionella pneumophila elaborates two aminopeptidases, as well as a metalloprotease that contributes to differential infection among protozoan hosts.. Appl Environ Microbiol.

[31] Sahr T, Bruggemann H, Jules M, Lomma M, Albert-Weissenberger C (2009). Two small ncRNAs jointly govern virulence and transmission in Legionella pneumophila.. Mol Microbiol.

[32] Faucher SP, Friedlander G, Livny J, Margalit H, Shuman HA (2010). Legionella pneumophila 6S RNA optimizes intracellular multiplication.. Proc Natl Acad Sci U S A.

[33] Rasis M, Segal G (2009). The LetA-RsmYZ-CsrA regulatory cascade, together with RpoS and PmrA, post-transcriptionally regulates stationary phase activation of Legionella pneumophila Icm/Dot effectors.. Mol Microbiol.

[34] Brantl S (2009). Bacterial chromosome-encoded small regulatory RNAs.. Future Microbiol.

[35] Barrick JE, Sudarsan N, Weinberg Z, Ruzzo WL, Breaker RR (2005). 6S RNA is a widespread regulator of eubacterial RNA polymerase that resembles an open promoter.. RNA.

[36] Sharma CM, Hoffmann S, Darfeuille F, Reignier J, Findeiss S (2010). The primary transcriptome of the major human pathogen Helicobacter pylori.. Nature.

[37] Oliver HF, Orsi RH, Ponnala L, Keich U, Wang W (2009). Deep RNA sequencing of L. monocytogenes reveals overlapping and extensive stationary phase and sigma B-dependent transcriptomes, including multiple highly transcribed noncoding RNAs.. BMC Genomics.

[38] Muto A, Ushida C, Himeno H (1998). A bacterial RNA that functions as both a tRNA and an mRNA.. Trends Biochem Sci.

[39] Rubin CJ, Thollesson M, Kirsebom LA, Herrmann B (2005). Phylogenetic relationships and species differentiation of 39 Legionella species by sequence determination of the RNase P RNA gene rnpB.. Int J Syst Evol Microbiol.

[40] Berghoff BA, Glaeser J, Sharma CM, Vogel J, Klug G (2009). Photooxidative stress-induced and abundant small RNAs in Rhodobacter sphaeroides.. Mol Microbiol.

[41] D'Auria G, Jimenez-Hernandez N, Peris-Bondia F, Moya A, Latorre A (2010). Legionella pneumophila pangenome reveals strain-specific virulence factors.. BMC Genomics.

[42] Papenfort K, Vogel J (2010). Regulatory RNA in bacterial pathogens.. Cell Host Microbe.

[43] Cirillo SL, Yan L, Littman M, Samrakandi MM, Cirillo JD (2002). Role of the Legionella pneumophila rtxA gene in amoebae.. Microbiology.

[44] Bardill JP, Miller JL, Vogel JP (2005). IcmS-dependent translocation of SdeA into macrophages by the Legionella pneumophila type IV secretion system.. Mol Microbiol.

[45] Jules M, Buchrieser C (2007). Legionella pneumophila adaptation to intracellular life and the host response: clues from genomics and transcriptomics.. FEBS Lett.

[46] Tesh MJ, Miller RD (1981). Amino acid requirements for Legionella pneumophila growth.. J Clin Microbiol.

[47] Segal G, Russo JJ, Shuman HA (1999). Relationships between a new type IV secretion system and the icm/dot virulence system of Legionella pneumophila.. Mol Microbiol.

[48] Sorek R, Cossart P (2010). Prokaryotic transcriptomics: a new view on regulation, physiology and pathogenicity.. Nat Rev Genet.

[49] Svenningsen SL, Tu KC, Bassler BL (2009). Gene dosage compensation calibrates four regulatory RNAs to control Vibrio cholerae quorum sensing.. EMBO J.

[50] Lenz DH, Mok KC, Lilley BN, Kulkarni RV, Wingreen NS (2004). The small RNA chaperone Hfq and multiple small RNAs control quorum sensing in Vibrio harveyi and Vibrio cholerae.. Cell.

[51] Kohler R, Bubert A, Goebel W, Steinert M, Hacker J (2000). Expression and use of the green fluorescent protein as a reporter system in Legionella pneumophila.. Mol Gen Genet.

[52] Moffat JF, Tompkins LS (1992). A quantitative model of intracellular growth of Legionella pneumophila in Acanthamoeba castellanii.. Infect Immun.

[53] Croucher NJ, Fookes MC, Perkins TT, Turner DJ, Marguerat SB (2009). A simple method for directional transcriptome sequencing using Illumina technology.. Nucleic Acids Res.

[54] Craig DW, Pearson JV, Szelinger S, Sekar A, Redman M (2008). Identification of genetic variants using bar-coded multiplexed sequencing.. Nat Methods.

