# Supplementary material for: Sequencing Illustrates the Transcriptional Response of Legionella pneumophila during Infection and Identifies Seventy Novel Small Non-Coding RNAs
Source: PLoS One. 2011 Mar 3;6(3):e17570. doi: 10.1371/journal.pone.0017570 (PMC3048289; doi:10.1371/journal.pone.0017570)
Supplement: Figure S1 — L. pneumophila growth curve in BYE broth and intracellular. (DOC) [file pone.0017570.s013.doc]

**Figure S1.**


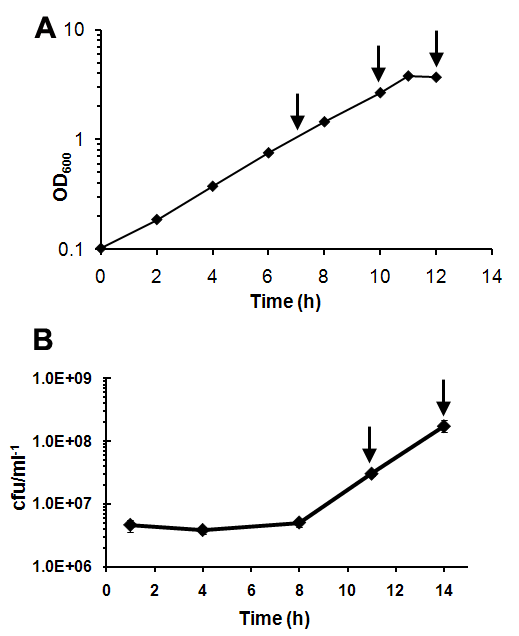


**Figure S1. Growth of *L. pneumophila* in BYE broth and intracellularly within *A. castellanii.***

A) Growth of of *L. pneumophila* Phil-1 in BYE broth at 37°C, 250 rpm. Bacterial growth was monitored at an optical density of 600 nm (OD_600_) after inoculation to an OD_600_ of 0.1. Time point t7 represents exponential growth (E), time point t10 late exponential growth (LE), time point t12 post-exponential growth (PE). Arrows indicate time points of sample collection.

B) Growth of *L. pneumophila* Phil-1 pMip.gfp inside of *A. castellanii* Neff. Bacterial growth was monitored by viable cell counts on BCYE plates. Time point t11 represents replicative phase (RP), t14 transmissive phase (TP). Shown are 2 biological replicates. Incubation temperature was at 37°C. Arrows denote time points of sample collection.
