# Supplementary material for: Sequencing Illustrates the Transcriptional Response of Legionella pneumophila during Infection and Identifies Seventy Novel Small Non-Coding RNAs
Source: PLoS One. 2011 Mar 3;6(3):e17570. doi: 10.1371/journal.pone.0017570 (PMC3048289; doi:10.1371/journal.pone.0017570)
Supplement: Figure S2 — GFP-expressing L. pneumophila inside of A. castellanii . (DOC) [file pone.0017570.s014.doc]

**Figure S2.**


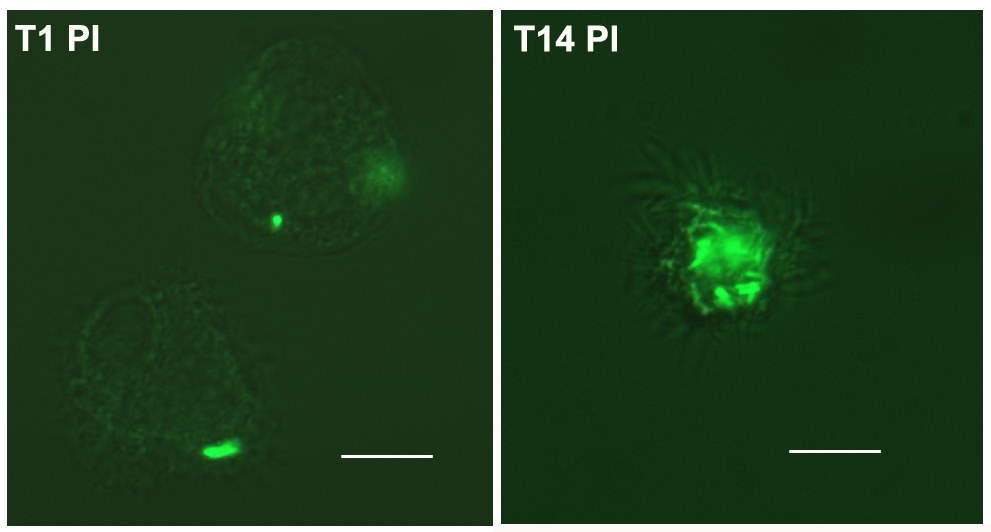


**Figure S2.** **Intracellular replication of GFP-expressing *L. pneumophila***.

Intracellular growth of *L. pneumophila* Phil-1 pMip.gfp inside of *A. castellanii* was monitored with a Zeiss Axioplan 2 fluorescence microscope after fixation of the cells in 1% Paraformaldehyde/PBS. Images were taken at1 hour (t1) and 14 hours (t14), post-infection (PI). Scale bar, 10 µm.
