# Supplementary material for: Sequencing Illustrates the Transcriptional Response of Legionella pneumophila during Infection and Identifies Seventy Novel Small Non-Coding RNAs
Source: PLoS One. 2011 Mar 3;6(3):e17570. doi: 10.1371/journal.pone.0017570 (PMC3048289; doi:10.1371/journal.pone.0017570)
Supplement: Figure S3 — Read coverage curve. (DOC) [file pone.0017570.s015.doc]

**Figure S3**


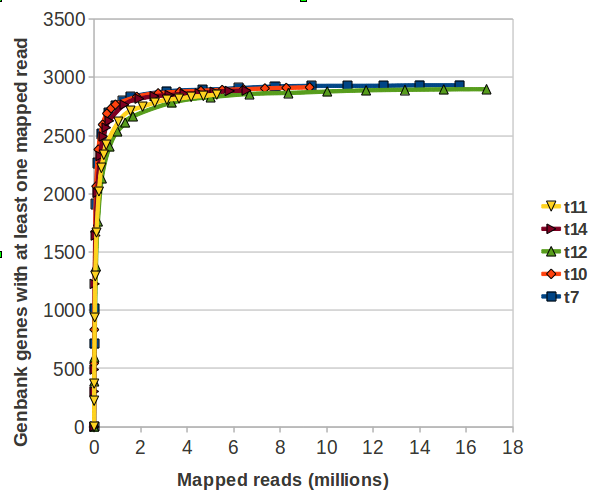


**Figure S3. Number of *L. pneumophila* Phil-1 genes in Genbank covered by at least one read at each time point.**

Gene coverage was assessed at various read numbers by artificially decreasing the total number of mapped reads and recalculating the number of genes covered. This graph highlights that sufficient coverage was obtained in this analysis to detect even lowly expressed transcripts and all time points plateaued at approximately the same level highlighting that the number of genes expressed across time points was relatively consistent. Growth time points of *L. pneumophila* Phil-1 grown in BYE broth are t7 (E), t10 (LE) and t12 (PE). Growth time points of *L. pneumophila* Phil-1 inside of *A. castellanii* Neff are t11 (RP) and t14 (TP).
