# Supplementary figures and images for: Sequencing Illustrates the Transcriptional Response of Legionella pneumophila during Infection and Identifies Seventy Novel Small Non-Coding RNAs
Source: PLoS One. 2011 Mar 3;6(3):e17570. doi: 10.1371/journal.pone.0017570 (PMC3048289; doi:10.1371/journal.pone.0017570)

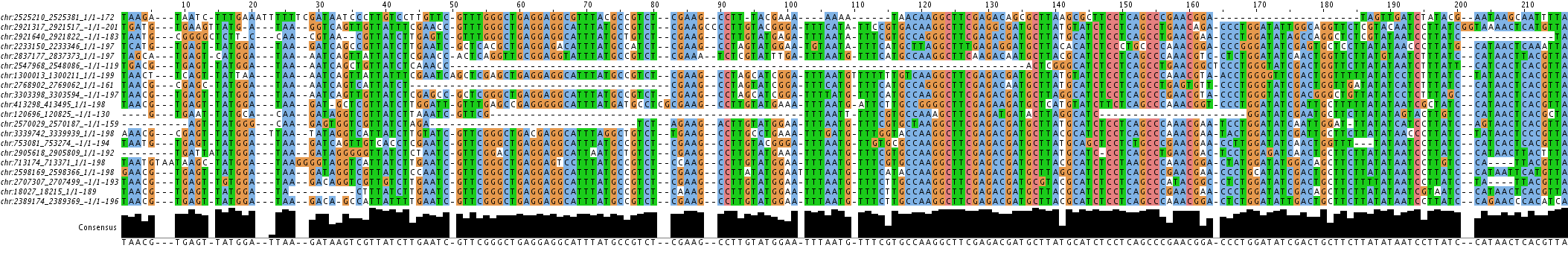


**Figure S4.** **Multiple sequence alignment of familyA non coding RNA members.**

Supplement: Figure S4 — Trimmed alignment of the non coding RNA family (family A). (DOC) [file pone.0017570.s016.doc]
